# Diffuse CD30‐Positive Cutaneous Infiltrate in a Clinically Suspected Hidradenitis Suppurativa Lesion: Histopathologic and Diagnostic Challenges

**DOI:** 10.1111/cup.70124

**Published:** 2026-04-22

**Authors:** R. Gervasi, G. L. Piazzetta, G. Soluri, C. Scigliano, C. Pelaia, V. Zuccalà, C. Gentile, N. Lobello, E. Allegra, E. Chiarella, N. Innaro

**Affiliations:** ^1^ UOC Endocrine Surgery, AOU Dulbecco Catanzaro Italy; ^2^ Otolaryngology Head and Neck Surgery University “Magna Græcia” Catanzaro Italy; ^3^ UOC General Surgery, AO San Giovanni di Dio Crotone Italy; ^4^ Department of Medical and Surgical Sciences University “Magna Græcia” of Catanzaro Catanzaro Italy; ^5^ Department of Human Pathology of Adult and Developmental Age “Gaetano Barresi,” Section of Pathology University of Messina Messina Italy; ^6^ Service's Department, Section of Anatomic Pathology A.O.U. Renato Dulbecco Catanzaro Italy; ^7^ Otolaryngology Department of Health Science Magna Graecia University of Catanzaro Catanzaro Italy; ^8^ Laboratory of Morphology and Tissue Cellular Biology, Department of Medical and Surgical Sciences Magna Graecia University of Catanzaro Catanzaro Italy

**Keywords:** ALK‐negative lymphoma, anaplastic large cell lymphoma, CD30‐positive lymphoma, cutaneous T‐cell lymphoma, hidradenitis suppurativa

## Abstract

Hidradenitis suppurativa (HS) is a chronic inflammatory skin disorder affecting apocrine gland–bearing areas. We report a 38‐year‐old male with a lesion in the left axilla, initially clinically interpreted as HS and resistant to antibiotics. Surgical excision and biopsy revealed a dense dermal and subcutaneous infiltrate of large atypical lymphoid cells with strong and diffuse CD30 expression (~80%–90% of tumor cells), CD4 positivity, ALK‐negativity, and loss of pan–T‐cell markers, consistent with anaplastic large cell lymphoma (ALCL). While the lesion was initially considered primary cutaneous, regional lymph node involvement and FDG‐avid sites on PET created diagnostic uncertainty regarding systemic disease. The patient received multi‐agent systemic chemotherapy followed by autologous stem cell transplantation, achieving complete metabolic remission. This case highlights the histopathologic challenges of diagnosing CD30‐positive lymphoid infiltrates in the context of chronic inflammatory skin disease, particularly when lesions are initially clinically misinterpreted as benign inflammatory conditions such as HS. Recognition of diffuse CD30 positivity, cytologic atypia, subcutaneous involvement, and loss of pan‐T‐cell markers is critical for distinguishing malignant from reactive lymphoid infiltrates. Early biopsy, immunophenotypic analysis, and clinicopathologic correlation are essential to guide appropriate management in atypical or treatment‐resistant lesions. Rather than demonstrating a novel association with HS, this report emphasizes diagnostic and classification challenges at the interface between primary cutaneous and systemic ALCL, which are of direct relevance to cutaneous pathologists.

## 1 Introduction

Hidradenitis suppurativa (HS) is a chronic, relapsing inflammatory skin condition that commonly affects areas like the armpits, groin, and perianal region. It presents painful nodules, recurrent abscesses, sinus tracts, and scarring, causing both physical and psychological burden [1]. HS affects 1%–4% of the population, is more common in females, and typically begins after puberty [1].

In addition to its skin‐related impact, HS is linked to an increased risk of malignancies. While squamous cell carcinoma (SCC) is the most commonly reported cancer, especially in chronic perineal lesions, recent studies also associate HS with a higher risk of hematologic cancers, such as non‐Hodgkin lymphoma, Hodgkin lymphoma, and cutaneous T‐cell lymphoma. This suggests that HS may predispose patients to a broader range of cancers beyond SCC [2, 3, 4].

The biological link between HS and cancer is not fully understood, but chronic inflammation is considered a key factor in cancer development. In HS, this involves persistent immune activation, oxidative stress, abnormal cytokine activity, and antigenic stimulation [5, 6]. The condition exemplifies systemic immune dysregulation, where abnormal immune responses, follicular blockage, and microbial imbalance lead to ongoing tissue damage and inflammation [7, 8].

Patients with HS have a significantly higher risk of developing lymphoma than the general population [2, 3, 9]. Because lymphoma can resemble or occur alongside HS inflammatory lesions, diagnosis is often delayed, making biopsy of persistent or unusual lesions essential [10].

This case highlights a rare but important diagnostic challenge, such as CD30‐positive cutaneous lymphomas, including anaplastic large cell lymphoma (ALCL), which may initially be clinically misinterpreted as chronic inflammatory conditions such as HS. It underscores the importance of maintaining a heightened index of suspicion for malignancy in patients presenting with atypical, progressive, or treatment‐resistant lesions. Prompt histopathologic evaluation is critical to differentiate benign inflammatory dermatoses from underlying neoplastic processes.

Although cases of CD30‐positive lymphomas arising in patients with HS have been previously reported, their classification and optimal management remain controversial. In particular, distinguishing primary cutaneous disease from early systemic involvement can be challenging when clinical, histopathological, and imaging findings are discordant. The present case illustrates these diagnostic uncertainties and how they may directly influence therapeutic decision‐making, including the choice of a systemic treatment approach.

## 2 Case Report

A 38‐year‐old male patient presented to the endocrine surgery outpatient clinic in September 2021 with a progressively worsening lesion in the left axillary region that had not responded to antibiotic treatment. The lesion had first appeared 6 months earlier and was initially interpreted as HS on clinical grounds, despite its atypical course and lack of response to antibiotic therapy. Based on this diagnosis, the patient underwent multiple courses of antibiotics and anti‐inflammatory therapies; however, no clinical improvement was observed, and the lesion continued to deteriorate over time.

Although initially diagnosed as HS, the lesion's subsequent clinical appearance—characterized by fungating and ulcerative changes—was atypical and inconsistent with classic HS presentations.

A culture of the lesion identified the presence of *Enterobacter cloacae* complex, likely reflecting a shift in the local bacterial flora due to antibiotic pressure on the typical Gram‐positive microbiota of the skin and soft tissues. Despite the initiation of broad‐spectrum antibiotics, the lesion remained unresponsive.

The patient was alert, cooperative, and without fever, with generalized skin ichthyosis but no major comorbidities.

The patient reported a 10‐year history of recurrent abscesses consistent with HS involving the groin and perianal regions. At the time of presentation, there were no active HS lesions in the axillary area or elsewhere. In addition, no sinus tracts, scarring, or other typical HS‐related skin changes were observed in the left axilla.

**FIGURE 1:**
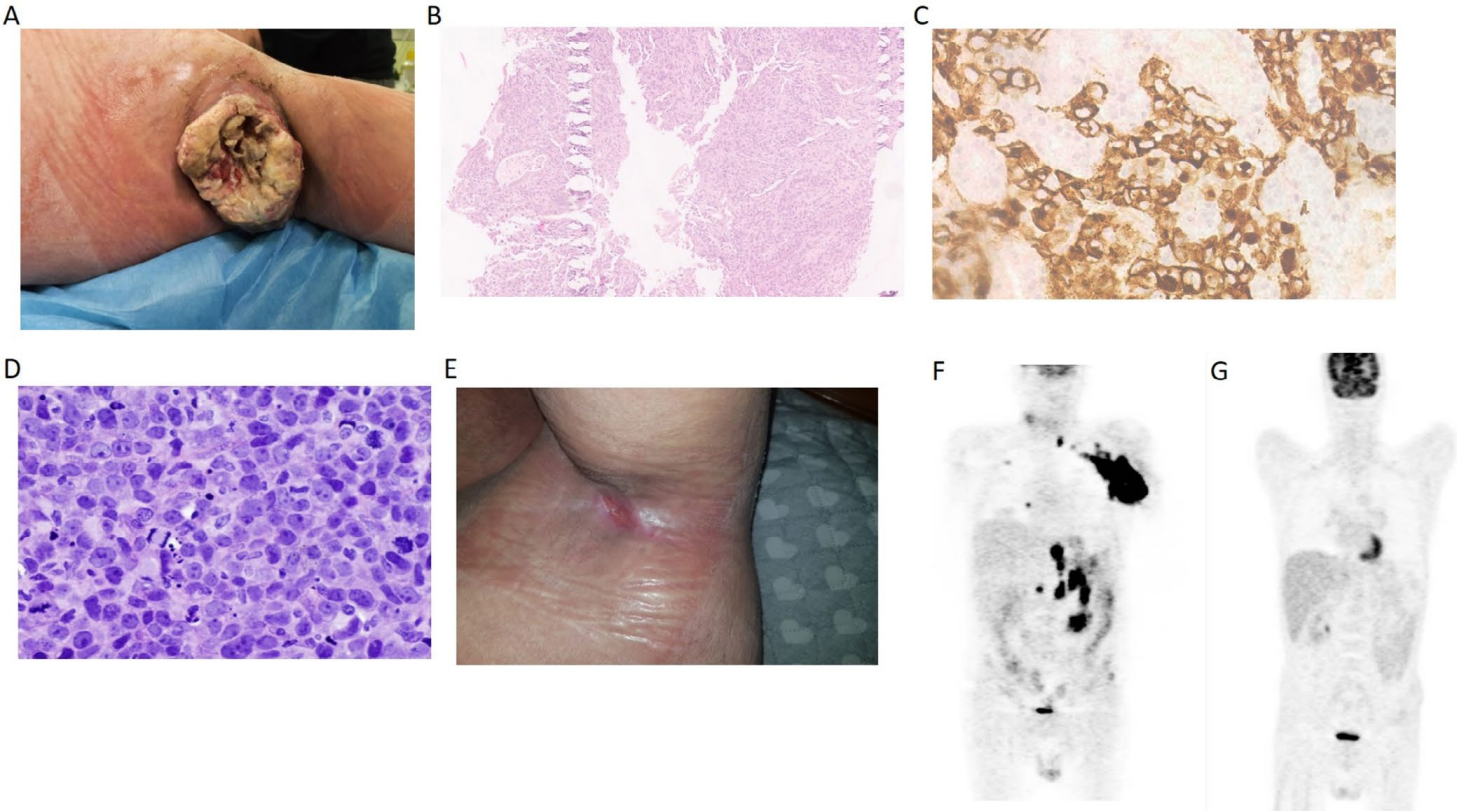
ALK‐negative anaplastic large cell lymphoma (ALCL) presenting as an axillary mass. (A) Clinical image of the left axillary region showing a large, ulcerated lesion with tissue loss, extensive edema, and impaired limb function. (B) Low‐power view demonstrating a dense dermal and subcutaneous infiltrate composed of atypical lymphoid cells with overlying epidermal ulceration (hematoxylin–eosin [H&E], original magnification 100×). (C) Immunohistochemical staining showing strong membranous and cytoplasmic CD30 positivity in large atypical lymphoid cells (immunohistochemistry for CD30, original magnification 400×). CD30 expression was diffuse and intense (> 80% of neoplastic cells), distinguishing the neoplastic infiltrate from the sparse reactive CD30‐positive lymphocytes occasionally observed in chronic inflammatory lesions of hidradenitis suppurativa. (D) High‐power view highlighting large pleomorphic lymphoid cells with abundant cytoplasm and prominent nucleoli (H&E, original magnification 400×). (E) Postoperative image showing vacuum‐assisted closure (VAC) therapy applied to the axillary wound. (F) Positron emission tomography (PET) scan demonstrating multiple areas of increased fluorodeoxyglucose (FDG) uptake, including sites distant from the primary axillary lesion, suggestive of possible extracutaneous involvement. (G) Follow‐up PET scan performed in May 2023 showing complete metabolic remission with no residual FDG‐avid lesions.

Examination showed a large, ulcerated lesion in the left armpit, significant tissue loss, extensive limb swelling, and limited arm movement (Figure 1A). Chest and abdominal exams were normal. Laboratory tests—including blood count, biochemical panel, ECG, and chest x‐ray—were all within normal limits. Serologic tests for hepatitis B, hepatitis C, and HIV were negative. The patient was not receiving systemic immunosuppressive therapy before diagnosis, and there was no history of corticosteroid or biologic use for HS. His prior treatments consisted solely of topical antiseptics and intermittent courses of oral antibiotics.

The lesion's rapid worsening, severe tissue damage, and lack of response to antibiotics were unusual for typical HS. The absence of common HS features like pus and sinus tracts, along with deep tissue involvement, suggested a different or underlying disease. It is important to note that the diagnosis of HS involving the axillary lesion was based solely on clinical assessment and was never histologically confirmed. In retrospect, the relentless growth of the lesion, the absence of hallmark features of HS, and the complete lack of response to antibiotic therapy strongly suggest that the initial diagnosis of HS was likely incorrect and that the lesion represented a neoplastic process from its onset. These warning signs led the medical team to perform surgery to treat the area and collect tissue for diagnostic analysis.

The patient's clinical course—from initial dermatology assessment and failed antibiotics to surgery and final diagnosis—highlights the importance of closely monitoring lesion progression. Microbiological tests, imaging, and surgical debridement all contributed essential information leading to the biopsy. This case underscores the value of multidisciplinary collaboration among dermatologists, surgeons, and pathologists for accurate diagnosis.

The patient underwent urgent surgical debridement of necrotic skin and subcutaneous tissue. Histopathological examination confirmed that the primary lesion was based in the dermis and subcutis, with overlying epidermal ulceration and no evidence of lymph node architecture, consistent with a skin‐based CD30‐positive ALCL, initially interpreted as primary cutaneous ALCL (pcALCL). Although one regional lymph node was excised for staging, no histological evidence of nodal disease was found. A firm lymph node adherent to adjacent structures was also excised during the procedure for diagnostic purposes; however, a full lymph node dissection was not performed.

Because the lesion progressed aggressively and did not respond to antibiotics, doctors expanded the differential diagnosis to include atypical infections (like cutaneous tuberculosis or deep fungal infections), neutrophilic dermatoses (e.g., pyoderma gangrenosum), and primary cutaneous lymphomas. This broader approach was important since overlapping symptoms can delay diagnosis without a biopsy.

Histopathological analysis revealed a dense dermal infiltrate composed of large atypical lymphoid cells with pleomorphic nuclei and a high mitotic index. The neoplastic infiltrate involved dermis and subcutis with overlying epidermal ulceration. Immunohistochemistry demonstrated strong and diffuse CD30 positivity in approximately 80%–90% of tumor cells, confirming the diagnosis of ALCL (Figure 1B,C). The tumor cells also expressed CD4, MUM1, and EMA, consistent with a T‐helper phenotype and activated lymphoid origin. Notably, there was no expression of pan‐T‐cell markers such as CD2, CD3, CD5, CD7, or CD8, indicating a loss of T‐cell antigen expression, which is common in ALCL. The absence of B‐cell markers (CD20, PAX5) further excluded a B‐cell lineage. Importantly, ALK1 was negative, establishing the lesion as an ALK‐negative variant of ALCL. A high Ki‐67 proliferation index (> 80%) was observed, consistent with the typically elevated proliferative activity seen in pcALCL, though not used for formal grading (Figure 1D and Table 1). In addition, infiltration of an adjacent lymph node was identified. In light of the nodal involvement, EMA positivity, and FDG‐avid extracutaneous sites on positron emission tomography (PET) imaging, a systemic or nodal ALCL with secondary cutaneous involvement could not be definitively excluded. The case was therefore considered to lie at the interface between primary cutaneous and systemic ALK‐negative ALCL. The main histological differential diagnoses included lymphomatoid papulosis (LyP), transformed mycosis fungoides, and reactive CD30‐positive dermatitis.

**TABLE 1:** Immunohistochemical profile of the axillary lesion diagnosed as primary cutaneous ALCL.

Marker	Expression
CD30	Positive
CD4	Positive
MUM1	Positive
EMA	Positive
CD20	Negative
CD3	Negative
CD8	Negative
CD2	Negative
CD7	Negative
CD5	Negative
PAX5	Negative
ALK1	Negative
Ki‐67	High (> 80%)

*Note:* Immunohistochemical characterization of the axillary lesion revealed a T‐helper phenotype, ALK‐negative anaplastic large cell lymphoma (ALCL). The expression profile shows strong positivity for CD30, CD4, MUM1, and EMA, while typical B‐ and T‐cell markers (CD20, CD3, CD8, CD2, CD7, CD5, PAX5) were absent. ALK1 negativity and a high Ki‐67 index are consistent with a diagnosis of primary cutaneous ALCL with aggressive features.

Postoperatively, the patient underwent vacuum‐assisted closure (VAC) therapy to promote wound healing in the axillary region (Figure 1E). For staging purposes, a PET scan was performed, which revealed increased metabolic activity limited to the skin and regional lymph nodes and additional sites distant from the primary axillary lesion. Although none of these extracutaneous sites were biopsied, their distribution raised concern for possible early systemic involvement and contributed to diagnostic uncertainty (Figure 1F).

The patient initiated an oncologic treatment protocol consisting of multiple lines of chemotherapy followed by autologous stem cell transplantation. Due to the lesion's aggressive behavior and equivocal PET findings suggestive of possible extracutaneous involvement, the oncology team proceeded with a systemic treatment strategy. Although bone marrow biopsy showed no systemic spread, the multidisciplinary team opted for chemotherapy and autologous stem cell transplantation out of caution. At the latest follow‐up (May 2023), the patient was clinically well, with complete wound healing and no cutaneous or systemic symptoms. A follow‐up PET scan demonstrated complete metabolic remission, with no residual FDG‐avid lesions (Figure 1G).

Informed consent for publication, including the use of clinical images and anonymized medical details, was obtained from the patient.

## 3 Discussion

Several studies have reported an increased incidence of lymphoma in patients with HS; however, this association should be interpreted with caution. Epidemiologic data suggest that patients with HS may have a twofold to fourfold increased relative risk of lymphoma compared to individuals without HS, yet the overall incidence remains low, and causality has not been established in mechanistic studies [11]. Moreover, observational associations do not prove that HS directly causes lymphoma, and confounding factors such as chronic inflammation and immune dysregulation may partly explain this relationship.

In many reported cases, the relationship may reflect diagnostic overlap rather than a true pathogenetic link. Aggressive cutaneous and systemic lymphomas may clinically mimic chronic inflammatory dermatoses such as HS, particularly in intertriginous areas, leading to initial misdiagnosis and diagnostic delay [2, 3]. A major limitation of this case is the absence of histological confirmation of HS in the axillary lesion. Although the patient had a remote clinical history suggestive of HS in other anatomical sites, the axillary lesion itself most likely represented lymphoma from its onset rather than malignant transformation of preexisting hidradenitis. This observation highlights that clinical features alone can be insufficient in distinguishing persistent inflammatory disease from masquerading malignancies when standard HS features are absent.

Patients with HS have an estimated two to four times higher relative risk of developing lymphoma compared to those without HS. However, this estimate derives from observational studies subject to bias, and the evidence base remains limited [3, 10].

While the absolute risk is low, the possibility of misdiagnosis or delayed diagnosis is clinically important because lymphomas—particularly T‐cell variants—can closely mimic chronic inflammatory dermatoses, leading to prolonged courses of inappropriate therapies [12].

In this case, the lesion was initially interpreted as treatment‐resistant HS because of its axillary location and the patient's prior history of HS in other anatomical regions. However, the lesion's clinical morphology—characterized by rapid growth, ulceration, and fungation—was not typical of classical HS. However, its clinical morphology—characterized by rapid growth, ulceration, and fungation—lacked hallmark HS features such as sinus tracts, purulent discharge, and multiple comedones. Its fungating, nodular appearance and rapid evolution were more consistent with a neoplastic process and, in retrospect, may have warranted earlier biopsy in clinical practice.

Chronic inflammation in HS not only drives the disease but may also promote cancer development. Repeated tissue damage and healing create a pro‐tumorigenic microenvironment through several mechanisms: ongoing immune activation causes genetic instability and uncontrolled cell growth; oxidative stress from reactive oxygen species leads to DNA damage; high levels of inflammatory cytokines (IL‐1β, IL‐6, TNF‐α) stimulate cell proliferation and tissue changes; and activation of cancer‐related pathways (NF‐κB, STAT3, MAPK) supports tumor growth and resistance to apoptosis [5, 6]. Although these mechanisms are biologically plausible, direct evidence linking HS‐associated inflammation to lymphomagenesis remains limited.

A useful comparison can be drawn with ALCLs arising in the setting of chronic antigenic stimulation related to foreign materials, most notably breast implant‐associated ALCL (BIA‐ALCL), and other rare device‐associated lymphomas. BIA‐ALCL is typically an ALK‐negative, CD30‐positive T‐cell lymphoma that develops in the fibrous capsule surrounding textured breast implants and is thought to result from prolonged immune stimulation, biofilm formation, and local cytokine dysregulation [13]. The average time from implant placement to clinical presentation is approximately 7–10 years, with most patients presenting with peri‐implant seroma and swelling [14]. Similar pathogenetic mechanisms have been proposed in rare ALCL cases associated with other prosthetic devices, including orthopedic implants, tissue expanders, and dental implants [15].

Beyond prosthetic devices, analogous mechanisms of chronic immune stimulation may occur in other contexts. For example, rare cases of ALK‐positive ALCL have been reported in patients with HS receiving immunomodulatory therapy such as adalimumab, suggesting that persistent immune dysregulation—whether due to foreign materials or pharmacologic immunosuppression—can create a microenvironment permissive for lymphoma development [16].

These device‐associated entities share with the present case the broader biological mechanism of chronic immune activation within a confined anatomical site, which may promote clonal T‐cell expansion. However, important differences exist. Device‐associated ALCLs arise in direct spatial relations to an identifiable foreign body and commonly present as delayed seroma or peri‐implant effusion, whereas in our patient, no implanted material was present, and the lesion developed in a region initially interpreted as inflammatory dermatosis. Moreover, epidemiologic and molecular data supporting a causal link between chronic inflammation and lymphomagenesis are stronger in device‐associated ALCL than in HS, where the relationship remains speculative and confounded by longstanding inflammation.

This comparison underscores a broader oncologic principle: persistent immune stimulation—whether driven by foreign materials, chronic infection, or inflammatory dermatoses—may create a permissive microenvironment for lymphomagenesis. Nevertheless, temporal or anatomical association alone should not be interpreted as evidence of causation in the absence of robust mechanistic data.

Interestingly, CD30‐positive lymphocytes can occasionally be found in chronic inflammatory dermatoses, including HS; however, in HS, these cells are typically sparse and represent reactive, activated lymphocytes in the context of chronic inflammation rather than neoplastic cells. CD30 is a marker that can be expressed on activated T cells in a variety of inflammatory conditions, and low‐level CD30 expression has been documented in reactive cutaneous immune infiltrates, supporting a polyclonal immune response rather than clonal proliferation [17].

Recent immunohistochemical studies in HS and other inflammatory disorders highlight that scattered CD30‐positive lymphocytes may be present within areas of chronic inflammation, where they reflect activation of T‐cell subsets rather than malignancy [18].

In contrast, CD30‐positive lymphoproliferative disorders such as pcALCL and LyP are defined by dense infiltrates of large atypical cells with strong and diffuse CD30 expression, accompanied by monoclonal T‐cell receptor gene rearrangements and characteristic immunophenotypic profiles. These entities are part of the primary cutaneous CD30‐positive T‐cell lymphoproliferative spectrum and require clinicopathologic integration for accurate diagnosis [19].

Recent comprehensive reviews of CD30‐positive primary cutaneous lymphoproliferative disorders emphasize that strong, diffuse CD30 positivity in the majority of tumor cells, often with associated loss of conventional T‐cell markers and expression of additional markers such as EMA and CD4, supports a monoclonal malignant process distinct from reactive inflammatory infiltrates [20].

This contrasts sharply with what is typically observed in nonmalignant HS lesions, where CD30 positivity—when present—tends to be limited to a small proportion of scattered reactive lymphocytes, and clonality studies generally reveal polyclonal T‐cell receptor rearrangements consistent with a reactive process rather than lymphoma [17].

Therefore, while CD30 positivity can occur in the inflammatory milieu of HS, the pattern (diffuse vs. scattered), density, cytologic features, and evidence of clonality of CD30‐positive cells are key distinguishing features separating reactive HS lesions from CD30‐positive lymphomas such as pcALCL. Immunophenotypic analysis, including CD30, clonality assessment, and additional markers, is therefore critical for accurate diagnosis when clinical or histologic features deviate from typical HS [17].

While chronic inflammation has been proposed as a potential risk factor for lymphoma development, there is no direct evidence that the patient's ALCL arose from preexisting HS. The lesion arose in an area previously affected by HS but lacked concurrent HS‐specific histological features, and no clear transitional zones between chronic inflammation and neoplastic transformation were identified.

Key clinical points from this case are: (1) persistent or unusual HS lesions warrant biopsy to exclude cancer; (2) clinicians should recognize that ALCL can rarely present like HS; and (3) immunohistochemistry is essential for diagnosis since ALCL may lose conventional T‐cell markers while expressing CD30, CD4, and EMA, with a high proliferation rate, making diagnosis challenging.

This case underscores the need for careful clinical observation and timely biopsy in patients with atypical HS, especially when lesions worsen or fail to respond to treatment. A comparative understanding of CD30 expression in inflammatory versus neoplastic lesions may further assist clinicians in distinguishing reactive HS from lymphoma in challenging cases. In this patient, disease progression and unusual biopsy findings ultimately led to the diagnosis of a CD30‐positive ALCL with predominant cutaneous presentation, although early systemic involvement could not be conclusively ruled out.

In conclusion, hematologic malignancies may be clinically misinterpreted as HS in their early presentation, leading to diagnostic delay and inappropriate initial management. Early biopsy of suspicious or treatment‐resistant lesions is critical for accurate diagnosis and improved patient outcomes. This case also underscores the limitations of current classification systems when clinical behavior, imaging findings, and histopathology are not fully concordant—for example, when extensive FDG uptake at distant sites may suggest early systemic disease despite criteria for pcALCL. In such borderline presentations, treatment decisions may reasonably err on the side of systemic therapy.

## Author Contributions

Conceptualization: G. Soluri, C. Scigliano, N. Innaro, E. Chiarella. Writing – original draft preparation: G. Soluri, C. Scigliano, N. Lobello, N. Innaro. Review and editing: E. Chiarella, G.L. Piazzetta, C. Pelaia, E. Allegra, C. Gentile, V. Zuccalà, C. Gentile. Funding acquisition: G. L. Piazzetta and C. Pelaia. All authors have read and approved the final manuscript.

## Funding

Open access funding provided by Università degli Studi Magna Graecia di Catanzaro within the CRUI‐CARE Agreement.

## Ethics Statement

The authors have nothing to report.

## Consent

Written informed consent to participate was obtained from the patient.

## Conflicts of Interest

The authors declare no conflicts of interest.

## Data Availability

The data that support the findings of this study are available from the corresponding author upon reasonable request.
